# Motivational brief intervention for the prevention of sexually transmitted infections in travelers: a randomized controlled trial

**DOI:** 10.1186/1471-2334-11-300

**Published:** 2011-11-01

**Authors:** Nicolas Senn, Serge de Valliere, Didier Berdoz, Blaise Genton

**Affiliations:** 1 Travel Clinic, Department of Ambulatory Care and Community Medicine, University of Lausanne, Switzerland; 2 Alcohol Treatment Centre, University of Lausanne, Switzerland; 3 Infectious Diseases Service, University Hospital, Lausanne, Switzerland; 4 Swiss Tropical and Public Health Institute, University of Basel, Switzerland

## Abstract

**Background:**

Sexually transmitted infections (STIs) are among the frequent risks encountered by travelers. Efficient interventions are needed to improve the understanding of the risks of STIs. We investigated the potential benefits of a motivational brief intervention (BI) and the provision of condoms on the engagement in unprotected casual sex.

**Methods:**

3-arm randomized controlled trial performed among single travelers aged 18-44 years visiting a travel clinic in Switzerland. The main outcomes were the prevalence of casual unprotected sexual intercourse and their predictors.

**Results:**

5148 eligible travelers were seen from 2006 to 2008. 1681 agreed to participate and 1115 subjects (66%) completed the study. 184/1115 (17%) had a casual sexual relationship abroad and overall 46/1115 (4.1%) had inconsistently protected sexual relations. Women (adjusted OR 2.7 [95%CI 1.4-5.6]) and travelers with a history of past STI (adjusted OR 2.8 [95%CI 1.1-7.4]) had more frequent casual sexual relationships without consistent protection. Regarding the effect of our intervention, the prevalence of subjects using condoms inconsistently was 28% (95%CI16-40) in the motivational BI group, 24% (95%CI10-37) in the condoms group and 24% (95%CI14-33) in the control group (p = 0.7).

**Conclusion:**

This study showed that a motivational brief intervention and/or the provision of free condoms did not modify risky sexual behavior of young travelers. The rate of inconsistently protected sexual relationships during travel was however lower than expected

**Trial Registration Number:**

ClinicalTrials.gov: NCT01056536

## Background

Among the risks frequently encountered by travelers abroad, sexually transmitted infections (STIs) such as HIV, hepatitis B, herpes simplex, syphilis or urethritis are a well recognized problem [1-3]. Epidemiological studies on the sexual behavior of travelers have shown that 20 to 50% of them will have sexual intercourse with occasional partners (local persons or other travelers) with a rate of condom use not exceeding 50-70% [1,3-8]. Some risk factors for unprotected sexual practices abroad have been identified, the most important ones being traveling without regular partner, expecting to have casual sex, casual sexual contacts in the home country, non-tourist journeys and recreational drug use [8].

With the emergence of the HIV/AIDS epidemic, important efforts have been made at both individual and population levels to promote safe sexual practices and the use of condoms through national prevention campaigns, distribution of condoms or personalized counseling [9]. Different interventions have been developed aiming to improve the understanding of the risks of STIs and the adherence to preventive measures. However, the rate of unprotected casual sex has remained unchanged in the last 10 years [10]. Although the prevalence of HIV/AIDS in western countries has been stable in the past few years, the incidence of STIs such as syphilis has increased dramatically (12 millions of new cases per year worldwide) [11]. To explain this lack of effectiveness, it has been postulated that these preventive strategies have usually only proved to be effective in the frame of well controlled trials, usually in specialized clinics, but have failed to show any benefit when applied to the "real world" [12].

There is therefore a need to develop new strategies to improve the adherence to preventive measures,[9] which are adapted to day-to-day clinical practice. If the content of the information is essential, the way to deliver it is also important. It is especially relevant in health promotion messages. Taking this into account, motivational interviewing was developed by Rollnick and Miller in the late 1980s and applied with success essentially in the treatment of problem drinkers [13]. According to these authors "motivational interviewing is a directive, client-centered counseling style for eliciting behavior change by helping clients to explore and resolve ambivalence" [14]. It has also been applied with success to other fields such as the management of diabetic patients in the general practice setting [15]. When health workers are using this approach with patients, it is essential that they understand the spirit of motivational interviewing. Derived from motivational interviewing, brief interventions (BI) are shorter and therefore easier to integrate in a standard medical consultation. They have been developed and have proved to be effective essentially in the prevention of excessive alcohol consumption [16]. This approach has been only rarely used in the prevention of STIs and usually only in the context of highly selected populations, such as patients of HIV clinics [17]. If this approach seems to be well suited for interventions that aim to prevent risky sexual behaviors during travel, its effectiveness in the "real world" context of a day-to-day travel clinic has never been investigated.

In the present study, we aimed to investigate prospectively in a travel clinic the impact of a motivational BI associated with the provision of free condoms on the prevalence of travelers engaging in unprotected sexual intercourse abroad with occasional partners. This intervention was compared to travelers receiving either free condoms or a standard pre-travel information only. In addition potential predictors of unprotected sex were looked for.

## Methods

### Design, population and site

A prospective 3-arm randomized controlled trial was conducted at the travel clinic, Department of Ambulatory Care and Community Medicine, University Hospital, Lausanne, Switzerland between January 2006 and December 2008. The travel clinic provides pre-travel consultations including immunization and individual counseling. The clinic is run by several nurse practitioners and senior medical officers under the supervision of travel medicine specialists. Clients who visit the clinic are taken in consecutive order by the clinic staff. A standard consultation lasts from 15 to 25 minutes. About 7000 new clients are seen yearly in the clinic.

Study participants were recruited by the administrative staff in the waiting room of the clinic and were eligible for the study if aged between 18 and 44 years and if they were planning to travel without their regular partner. The study was always introduced using the same wording to avoid bias by giving too much uncontrolled information about STIs. In brief, they were asked if they were willing to participate in a study investigating the risk of STIs abroad. If they agreed, they had to fill a pre-travel questionnaire on their life habits at home and previous trips abroad. They were informed that they would receive a post-travel postal questionnaire. Then, they were randomly assigned to one of the 3 arms of the study: 1) Standard pre-travel consultation 2) Standard pre-travel consultation plus provision of free condoms or 3) Standard pre-travel consultation, motivational BI and provision of free condoms. Two weeks following their return, the post-travel questionnaire was sent to them.

### Interventions

Two different intervention arms and one controlled arm were established:

#### 1. Motivational BI and free condom provision

Four clinic staff (3 nurses and 1 medical officer) were trained to perform motivational BI (see below). When seeing a study participant, they performed a standard pre-travel consultation and then asked permission to talk for a couple of minutes about STIs. If the study participant agreed, a complete motivational BI on STIs was conducted and a free box of 3 condoms was offered.

The motivational BI used for the present study was developed in collaboration with the Alcohol Treatment Centre, University Hospital, Lausanne, Switzerland, which has conducted extensive research in this field [18]. The motivational BI was semi-structured and composed of 6 items summarized in table 1. The main focus was to explore the ambivalence of the traveler to change his sexual behavior and to adopt a safer attitude in the event of a casual sexual relationship while travelling. It was especially designed to fit in a pre-travel consultation, and lasted about 5 minutes. The information on STIs delivered during the motivational BI was standardized and summarized on an information sheet delivered to the traveler. It was divided in three topics: 1) prevalence of sexual intercourse and rate of condom use while traveling, 2) general information on the different STIs and prevalence rates around the world, 3) the different means of protection against STIs. Travelers were offered a free box of 3 condoms for their trip at the end of the motivational BI. Before the study started, motivational BI feasibility was tested during role-play sessions. All medical staff using this intervention underwent a half day of sensitization to the motivational interviewing technique and basic training at the beginning, as well as an annual update of 2 hours. They were asked not to share the content of the motivational BI with staff members assigned to other study arms.

**Table 1:** Structure of motivational Brief Interventions for the prevention of STIs in travelers

1	Ask the traveler's permission to talk about life habits and sexual behaviour during travel
**2**	Give information about STIs

**3**	Ask the traveler's opinion about the information presented

**4**	Ask the traveler's opinion on STIs and his upcoming trip

**5**	Explore the inconveniences and advantages (ambivalence) of having protected sexual intercourse during travel and summarise.

**6**	Propose a free box of 3 condoms.

#### 2. Free condom provision

Clients underwent a standard pre-travel consultation as described above. In addition the health care worker had to mention that there is a risk of STIs in case of unprotected sexual intercourse and a free box of 3 condoms was offered.

#### 3. Control arm

Clients underwent a standard pre-travel consultation as it is performed in the clinic, with no specific instructions given to the health care workers in regards to STI prevention, but knowing that all staff members are advised to provide travelers with standard information developed by the national department of public health on this topic.

### Outcomes

The primary outcome measure was the overall proportion of travelers aged 18-44 years traveling without their regular sexual partner and engaging in unprotected sexual intercourse abroad with a new partner in the 3 different treatment arms. The secondary outcomes were the proportion of any sexual intercourse abroad with a new partner in travelers aged 18-44 years traveling without their regular sexual partner and the predictors of any and unprotected sexual intercourse abroad with a new partner. Sub-analysis also investigated primary and secondary outcomes according to gender.

### Sample size calculation

Based on an expected reduction of the prevalence of unprotected intercourse abroad of 30% (15% to 10%), a power of 80% and a significance of 0.05, we calculated an original sample size of 726 travelers per arm for a total of 2178. However, due to logistical constraints and a recruitment rate slower than expected, an interim analysis was performed after 3 years and the study stopped due to absence of impact of any of the interventions.

### Randomization and blinding

All participants were randomly assigned by order of arrival by the administrative staff to one of the clinical staff members trained in one of the 2 study interventions or a member of staff without special training (control arm). Neither the study participants nor the study staff members were blinded due to the nature of the intervention.

### Data collection and analysis

All data were collected using self-administered questionnaires. Data were entered in Access software (Microsoft Office 2003). Multivariate analysis for predictors of unprotected sexual intercourse was performed on STATA software (version 10.0) using a logistical regression model.

The futility analysis was performed according to the model developed by Lachin *et al*[19].

### Ethics

The protocol was approved by the ethical committee of the University of Lausanne in September 2005.

## Results

**Figure 1:**
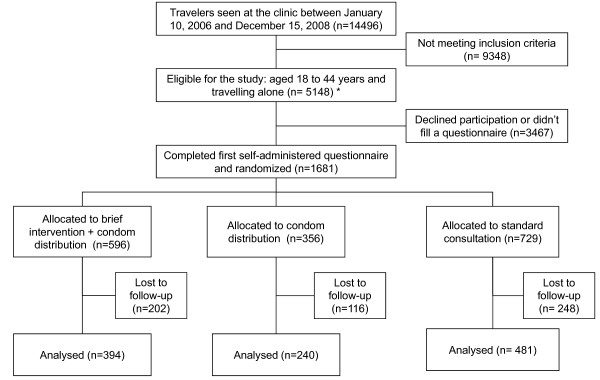
**Flow diagram of the study**.

Between January 2006 and December 2008, 5148 eligible travelers were seen at the Travel Clinic. 1681 travelers accepted to complete the pre-travel questionnaire. Figure 1 shows the flow diagram of the study.

596 travelers were randomly allocated to the brief intervention group, 359 to the condoms group and 729 to the control group. The numbers differ from one group to the other since the staff trained for the two interventions were not always present in the travel clinic (some were working part-time) although the study was going on every working day.

1115 subjects (66%) returned the post-travel postal questionnaire. There were no difference in the baseline characteristics between the participants who sent back the post-travel postal questionnaire and those who did not (data not shown). The proportion of subjects who completed the post-travel questionnaire was similar in all three allocation groups (66% in motivational BI group, 67% in condom group and 65% in control group).

In the motivational BI group, 77% (302/394) underwent a complete brief intervention as planned in the protocol. The reasons for not performing a motivational BI were recorded as follows: 36 subjects (40%) had no time, 15 subjects (16%) refused to talk about STIs, for 8 cases (9%) the health care worker forgot to do the motivational BI, and for 32 subjects (35%) the reason was not specified. In the condom group, 86% (207/240) accepted the box of condoms.

Table 2 shows the baseline characteristics of the participants. There was no statistical evidence for differences between all groups except for a history of previous STI. In the control group there were significantly more participants than in the condom and motivational BI groups who had had an STI in the past (respectively 12%, 4% and 6%; p = .002).

**Table 2:** Baseline characteristics of participants by intervention group (pre-travel questionnaire)

Characteristics	Brief intervention + condoms	Condoms only	Standard consultation group	p
n	394	240	481	
Mean age	29	29	29	
Women	210 (53%)	113 (47%)	262 (54%)	ns
Swiss or European nationals	367 (93%)	223 (95%)	448 (93%)	ns
Not married	347 (88%)	208 (87%)	424 (88%)	ns
No stable partner	199 (51%)	103 (43%)	230 (48%)	ns
**Habits at home**				

Alcohol use (one glass or more per week)	218 (55%)	132 (55%)	277 (58%)	ns
Tobacco use	116 (29%)	66 (28%)	125 (26%)	ns
Recreational drug use	40 (10%)	26 (11%)	54 (11%)	ns
Previous STI	23 (6%)	9 (4%)	56 (12%)	0.002
Previous HIV test	216 (56%)	143 (60%)	276 (57%)	ns
Casual sexual relationship in last 6 months	136 (35%)	92 (38%)	151 (31%)	ns
**For the trip to come**				

Considering likely to have new sexual relationship	54 (14%)	35 (15%)	74 (15%)	ns
Intention to take condoms	169 (43%)	104 (43%)	210 (44%)	ns

Among the 1115 subjects who returned the post-travel questionnaire, 184 (17%) had a casual sexual relationship during their trip. By univariate analysis, several characteristics were found to be predictors of having a new sexual relationship during the trip (table 3). By multivariate logistic regression analysis, the following characteristics remained associated with having casual sex abroad: planning to have sex (OR 5.8 [95%CI 3.7-9.0]), planning to take condoms (OR 2.5 [95%CI 1.6-3.9]), having had sex with a casual partner in the past 6 months (OR 1.8 [95%CI1.2-2.7]), using recreational drugs at home (OR 1.7 [95%CI 1.0-2.9]) and drinking alcohol at home (OR 1.6 [95%CI 1.1-2.3]) (table 3). Of the 184 subjects who had a sexual relationship during the current trip 46 (27%) used condoms inconsistently. In other words the overall rate of inconsistently protected new sexual relationships abroad was 4.1% (46/1115) (table 4). By multivariate regression analysis there were two predictors for new sexual relationships without consistent protection: female sex (adjusted OR of 2.7 [95%CI 1.4-5.6]) and a history of STI in the past (adjusted OR of 2.8 [95%CI 1.1-7.4]) (table 3).

**Table 3:** Predictors of having a new sexual relationship and unprotected sex during the trip (post-travel questionnaire)

		**New sexual relationship during trip**			**Unprotected sexual relationship during trip**		
	
	**n**	**Had sex with new partner**	**Crude OR (95%CI)**	Adjusted OR (95%CI)*	**Had unprotected sex**	**Crude OR (95%CI)**	Adjusted OR (95%CI)*
	
Sex (female)	594	84 (14%)	0.7 (0.5-0.9)	n/a	30 (36%)	2.9 (1.4-6.3)	2.7 (1.4-5.6)
Planning to have sex	162	92 (57%)	11.8 (9.1-17.2)	5.8 (3.7 - 9.0)	25 (27%)	1.2 (0.6-2.6)	n/a
Intention to take condoms	483	145 (30%)	6.3 (4.4-9.2)	2.5 (1.6 - 3.9)	33 (23%)	0.6 (0.3-1.4)	n/a
Casual sex in last 6 months	383	117 (31%)	4.2 (3.0-5.8)	1.8 (1.2 - 2.7)	31 (27%)	1.2 (0.6-2.7)	n/a
No stable partner	574	135 (24%)	3.0 (2.1-4.3)	1.2 (0.8 - 1.8)	33 (24%)	0.9 (0.4-2.1)	n/a
Drugs at home	119	38 (32%)	2.7 (1.8-4.2)	1.7 (1.0 - 2.9)	11 (31%)	1.3 (0.5-3.0)	n/a
Alcohol at home	632	132 (21%)	2.1 (1.5-3.0)	1.6 (1.1 - 2.3)	34 (26%)	1.2 (0.5-2.7)	n/a
Tobacco at home	312	76 (24%)	2.0 (1.5-2.8)	1.2 (0.8 - 1.8)	21 (28%)	1.3 (0.6-2.6)	n/a
Had STI in the past	89	21 (24%)	1.6 (0.9-2.7)	n/a	10 (48%)	3.2 (1.1-9.0)	2.8 (1.1-7.4)

* multivariate logisitc regression model including all variables with singificant OR's in univariate analysis

**Table 4:** Proportion of subjects who had new sexual relationships and proportion of travelers who used condoms inconsistently according to randomization group during previous and current travel

		**Previous travel**			**Current travel**		
		
	**N**	**New sexual relation**	**Rate of inconsistently protected sex**	**Inconsistent protection among travelers with new sexual relation**	**New sexual relation**	**Rate of inconsistently protected sex**	**Inconsistent protection among travelers with new sexual relation**
	
**Standard consultation**	481	61 (13%)	14/481 (2.9%)	14/61 (23%)	85 (18%)	20/481 (4.2%)	20/85 (24%)*
**Condoms only**	240	37 (15%)	10/240 (4.2%)	10/240 (27%)	42 (18%)	10/240 (4.2%)	10/42 (24%)*
**Brief intervention**	394	56 (14%)	11/394 (2.8%)	11/394 (20%)	57 (14%)	16/394 (4.1%)	16/57 (28%)*
**Total**	1115	154 (14%)	35/1115 (3.1%)	35/154 (23%)	184 (17%)	46/1115 (4.1%)	46 (25%)

*p = 0.42 by chi-square test

Looking at the effect of our interventions the proportion of subjects using condoms inconsistently was 28% (95%CI 16-40.) in the motivational BI group, 24% (95%CI 10-37) in the condom group and 24% (- 95%CI14-33) in the control group (p = 0,42) (table 3). Stratification by history of previous STI did not show any benefit of the interventions neither. For subjects who didn't report any past STI, the proportion using protection inconsistently for new sexual relations was 27% in the motivational BI group, 20% in the condom group and 19% in the control group (p = 0.5 by Pearson chi^2 ^test). For patients who reported a history of STI in the past, the proportion of inconsistently protected sex was 33% in the motivational BI group, 0% in the condom group and 46% in the control group (p = 0.26 by Pearson chi^2 ^test) For women we observed a trend for sexual intercourse to be less frequent in the motivational BI group (10% vs. 16%, p = 0.13) and a non-significant lower rate of unprotected sex (33% in the motivational BI group vs 36% in the two other arms).

The futility analysis performed at the end of the trial showed that the conditional power to detect a significant difference between the groups after recruitment of the initially planned 2178 subjects would have been 0.3% if the current trends were maintained. The conditional power would have increased to 21.5% if the further data had followed the initial hypothesis (reduction of 30% of the prevalence of unprotected intercourse abroad).

## Discussion

Young adults who are planning to travel without their regular sexual partner have been shown previously to be at particular risk for unprotected sexual relationships during their trip. It is therefore widely advocated that counseling about STIs should be part of the pre-travel consultation for such persons. There are however no studies which showed that such interventions are beneficial in reducing the rate of travelers who practice unprotected sex.

To evaluate if motivational BI could reduce the incidence of risky sexual behavior of travelers, we compared the rate of travelers engaging in unprotected sex among three different groups. The first group received a brief intervention on STIs and was provided with condoms. The second group was provided with condoms only and the third group had a standard pre-travel consultation performed where STI prevention was left to the discretion of the staff. This randomized controlled study failed to show any benefit of brief intervention in reducing risky sexual behavior compared to the other types of consultation (provision of condoms only or standard pre-travel consultation). In all three groups the proportion of subjects who had sexual intercourse during travel was similar (16 - 17%) and the proportion of subjects who used protection inconsistently for sexual intercourse varied between 28% in the motivational BI group, 24% in the free condom group and 25% in the control group, all differences being not significant.

Why did the motivational IBs not improve the rate of protection during casual sexual relationships? First, it is possible that the method of motivational BI is not more effective in real world settings than any other intervention on the same topic, such as for example providing condoms alone or performing a standard pre-travel consultation. Second, the subjects received the motivational BI in addition to all pre-travel advice. During travel clinic consultations the patients are given many recommendations on travel-related health risks; adding information on STIs might have been just too much for the patients to integrate. Last, there might have been a selection bias. Indeed, we conducted this intervention in a population attending a travel clinic, and thus more aware of health risks and less likely to engage in risky sexual relationships that the average traveler. In addition only one third of eligible subjects accepted to participate in the study. It is possible that those who accepted were made of a population more interested in the topic and being again at lower risk of sexual relations at risk. This is confirmed by a low rate of unprotected casual intercourse in our cohort (well below 30%) in both previous and current travel. It can therefore be postulated that improving the rate of protection becomes more difficult as we might have reached an awareness limit.

In our cohort, women were less likely to have sex while traveling (14% vs. 20% in males), but at the same time had a higher rate of unprotected sexual intercourse (36% in women vs 26% in men). This is consistent with the findings of Croughs et al showing that casual sex was more often unexpected in women than in men [8], thus reducing thus the chance for women to have condoms with them. This highlights the importance of specifically targeting women traveling alone with discussion of preventive measures. Interestingly a history of past STI was also associated with a lower rate of protected sex. This suggests that certain subjects have a different risk perception. Even a negative experience such as suffering from a STI in the past doesn't necessarily modify behaviors.

### Strengths and limitations

One could argue that our sample size of 1115 subjects was too small to demonstrate effectiveness of the motivational BI in reducing unprotected sex. We decided to stop the recruitment before we reached the estimated sample size of 2178 subjects, because of slow recruitment. An interim analysis showed at that stage an absence of any benefit of the tested intervention Furthermore, the ad hoc futility analysis performed at the end of the trial showed that the chance to detect any difference between the three treatment arms was below 1% if the current trends of low rate of unprotected sex intercourse were maintained. We did not record the number and details of the subjects who refused to participate in the study. We might have selected persons who were interested in the topic and who were already knowledgeable about the topic. Other people might exhibit more risky behavior.

A complete motivational BI was done for only 76% of subjects allocated to this group. The most frequent reason for not doing the intervention was the refusal of the patient. The baseline characteristics and the sexual behavior were however similar among the refusers and the subjects who accepted the intervention. However we can't exclude that this significant refusal rate reduced the effect of the intervention.

## Conclusions

In this study a motivational BI was not more effective in modifying risky sexual behavior of young travelers compared to standard pre-travel consultation or the provision of condoms. Further studies are needed to explore the benefits of prevention interventions in specific high risk groups, such as women and travelers with past history of STI.

### Ethical approval

The study protocol was approved by the Cantonal Ethics Committee on Human Research, Lausanne, Switzerland

## Competing interests

The authors declare that they have no competing interests.

## Authors' contributions

NS, DB and BG designed the study and wrote the study protocol. SV collected the data and supervised the overall conduct of the study. NS and SV did the analysis and wrote the first draft of the paper. All authors were involved with the interpretation, critical review of the paper, and gave final approval of the manuscript. NS and SV were the principal investigators.

## Pre-publication history

The pre-publication history for this paper can be accessed here:

http://www.biomedcentral.com/1471-2334/11/300/prepub
